# The First Pilot Genome-Wide Gene-Environment Study of Depression in the Japanese Population

**DOI:** 10.1371/journal.pone.0160823

**Published:** 2016-08-16

**Authors:** Takeshi Otowa, Yoshiya Kawamura, Akizumi Tsutsumi, Norito Kawakami, Chiemi Kan, Takafumi Shimada, Tadashi Umekage, Kiyoto Kasai, Katsushi Tokunaga, Tsukasa Sasaki

**Affiliations:** 1 Graduate School of Clinical Psychology, Teikyo Heisei University Major of Professional Clinical Psychology, Tokyo, Japan; 2 Department of Neuropsychiatry, Graduate School of Medicine, The University of Tokyo, Tokyo, Japan; 3 Department of Psychiatry, Shonan Kamakura General Hospital, Kanagawa, Japan; 4 Department of Public Health, Kitasato University School of Medicine, Sagamihara, Kanagawa, Japan; 5 Department of Mental Health, Graduate School of Medicine, The University of Tokyo, Tokyo, Japan; 6 Department of Human Genetics, Graduate School of Medicine, The University of Tokyo, Tokyo, Japan; 7 Laboratory of Health Education, Graduate School of Education, The University of Tokyo, Tokyo, Japan; College of Agricultural Sciences, UNITED STATES

## Abstract

Stressful events have been identified as a risk factor for depression. Although gene–environment (G × E) interaction in a limited number of candidate genes has been explored, no genome-wide search has been reported. The aim of the present study is to identify genes that influence the association of stressful events with depression. Therefore, we performed a genome-wide G × E interaction analysis in the Japanese population. A genome-wide screen with 320 subjects was performed using the Affymetrix Genome-Wide Human Array 6.0. Stressful life events were assessed using the Social Readjustment Rating Scale (SRRS) and depression symptoms were assessed with self-rating questionnaires using the Center for Epidemiologic Studies Depression (CES-D) scale. The p values for interactions between single nucleotide polymorphisms (SNPs) and stressful events were calculated using the linear regression model adjusted for sex and age. After quality control of genotype data, a total of 534,848 SNPs on autosomal chromosomes were further analyzed. Although none surpassed the level of the genome-wide significance, a marginal significant association of interaction between SRRS and rs10510057 with depression were found (p = 4.5 × 10^−8^). The SNP is located on 10q26 near *Regulators of G-protein signaling 10* (*RGS10*), which encodes a regulatory molecule involved in stress response. When we investigated a similar G × E interaction between depression (K6 scale) and work-related stress in an independent sample (n = 439), a significant G × E effect on depression was observed (p = 0.015). Our findings suggest that rs10510057, interacting with stressors, may be involved in depression risk. Incorporating G × E interaction into GWAS can contribute to find susceptibility locus that are potentially missed by conventional GWAS.

## Introduction

Depression is one of the world’s leading causes of morbidity. Because of the high prevalence of depression around the world, varying typically from 8% to 12% [1], the World Health Organization has predicted that it would rank second to ischemic heart disease in global burden of disease by 2020 [2]. Depression is a complex disorder that results from both genetic and environmental influences. Family and twin studies have shown that genetic factors are important in moderating the vulnerability to depression and heritability is estimated to range from 31% to 42% [3]. A large number of candidate gene studies of depression have been conducted. Suggestive evidence has been reported for associations between depression and candidate genes regulating the serotonin system (e.g., 5-HTTLPR, a functional polymorphism in the promoter region of the serotonin transporter gene *SLC6A4*), the hypothalamic-pituitary-adrenal (HPA) axis (e.g., *CRHR1*), or the neurotrophins axis (e.g., *BDNF*) [4, 5]. Recently, genome-wide association studies (GWAS) have suggested several single nucleotide polymorphisms (SNPs) associated with major depression [6–9] and depressive symptoms [10]. However, these candidate gene approach and GWAS have produced conflicting results for susceptibility genes for depression [11].

Stressful life events (SLEs) have been identified as a risk factor for depression. Several lines of studies have shown that SLEs are associated with the onset of depressive episodes and have shown a dose–response relationship [12]. Both recent and distal stressors (e.g., childhood adverse events) are associated with depression and their effects are additive [13]. Although distal stressors may have strong effect on psychopathology throughout the life course [14], the effects of stressors decrease with time and the most recent stressors have the greatest impact [15]. Therefore, the most recent SLEs (e.g., within 12 months) may be of direct etiological relevance to the onset of depression compared with distal events (>5 years before) [15–17]. For examples, Bebbington et al. investigated the effect of SLEs on the onset of depression and suggested that long-lasting effects of stressful events is less important than the effects of life events close to onset [15]. Kendler et al. found that the association between SLEs and depressive onsets was usually strongest in the month of occurrence but may extend for up to 6 months [16]. Rojo-Moreno et al. reported that depressive patients tended to experience more depressive provoking events than controls in the 12 months prior to the onset of the symptoms [17].

Given that only a portion of subjects exposed to SLEs develop depression, individual differences in genetic vulnerability to stressful events play a role in the development of depression. This genetic vulnerability is conceptually consistent with a gene × environment (G × E) interaction, in which specific alleles or genotypes are more or less sensitive to the effects of certain environmental exposure. To date, most G × E association studies of depression have focused on a limited set of biological candidate genes [18, 19]. To our knowledge, no genome-wide search for G × E interaction in depression has been published. Incorporating G × E interactions in GWAS is expected to increase the power to detect novel genes relevant to psychopathology [20]. Furthermore, dichotomization (i.e., case–control) and even sampling from the extremes of a distribution may be problematic because “healthy controls” may include depressive cases due to the high prevalence of depression, which decreases the statistical power [21]. Using continuous measure of depression (depressive symptoms) has been shown to greatly increase the power to detect genetic variants [22]. Thus, combining G × E interactions with GWAS data using continuous traits may be useful for discovering novel genetic variants [23].

The aim of the present study is to identify genes that influence the association of SLEs with depressive symptoms, performing a genome-wide G × E interaction analysis in the Japanese population. Among SLEs in adults, there is considerable evidence that work-related stress appears to precipitate depression in healthy workers [24]. Using a top finding from the results of the genome-wide G × E interaction analysis, we further investigated a similar G × E interaction between the genetic variant and work-related stress in an independent sample.

## Materials and Methods

### Ethics Statement

The study was approved by the Ethical Committees of the Kitasato University School of Medicine and the Graduate School of Medicine at the University of Tokyo. After complete description of the study to the subjects, written informed consent was obtained from all participants.

### Participants

All participants were genetically unrelated, nonclinical, Japanese corporation white-collar workers. For GWAS, 320 subjects (163 males and 157 females) were recruited in 2008 in Kanagawa Prefecture, adjacent to Tokyo, Japan. For a similar G × E interaction analysis in an independent sample, 439 subjects (276 males and 163 females) were recruited from an independent company in 2013 in Fukuoka Prefecture, located in the northern shore of Kyushu Island, Japan. These samples were described in detail elsewhere [25, 26]. The two areas are found to belong to the main cluster of the Japanese population [27]. The participants underwent a short structured diagnostic interview, the Mini-International Neuropsychiatric Interview (MINI) [28], or responded to questionnaires to rule out a current or past diagnosis of psychotic disorders.

### Measures

#### Stressful events

For the GWAS stage of the study, SLEs were assessed using the Social Readjustment Rating Scale (SRRS), which is a major life events inventory developed by Holmes and Rahe [29]. SRRS has been widely used in studies of psychosocial stress and illness, and includes 43 life events, each scored from 0 to 100 units of life change (ULC). The subjects were asked to rate which life event (e.g., “death of a spouse,” “divorce,” “marital separation,” “personal injury or illness,” “fired at work,” etc.) had occurred over the previous 12 months. The total score summing the values of the 43 life events was used in the analyses. A score ranging from 0 to 149 is defined as not associated with significant stress problems; a subject scoring 300 or higher is considered to be under major stress and to have an 80% chance of illness or health change [29].

For the replication stage of the G × E interaction analysis between top genes of GWAS and work-related stress in an independent sample set of 439 subjects, a stressful event was assessed by a self-administered question: “How many times have you been in fear of being unemployed in the last year?” The subjects could choose among the following answers: “1. none,” “2. once,” “3. more than once,” and “4. always.”

#### Psychological assessments

For the GWAS stage, depressive symptoms were assessed with a self-rating questionnaire using the Japanese version of the Center for Epidemiologic Studies Depression Scale (CES-D) [30], originally published by Radloff [31]. The questionnaire has been widely used to measure depressive symptoms in community populations and is also used as a screening tool for major depression [32]. CES-D is a 20-item, self-reported scale that focuses on depressive symptoms that occurred during the week prior to the questionnaire. The maximum score is set at 60, and higher scores correspond to more severe depressive symptoms. CES-D scores of 16 or higher indicate clinically relevant depressive symptoms, including both minor or subthreshold depression and major depression.

For the replication stage, depressive symptoms were assessed using the self-administered version of the K6 scale, which is generally used to screen subjects with depression on a large scale [33]. The participants were asked to answer six questions about how frequently they experienced symptoms of depression and anxiety during the past 30 days. The screening performance and acceptability of the Japanese versions of K6 has been validated [34]. To identify subjects at high risk of depressive symptom with K6, a cutoff of ≥9 was suggested according to a validation report, in which sensitivity and specificity were estimated at 77.8 and 86.4, respectively, in the Japanese population [35]. We used K6 scores as continuous variables in the present study.

### Genotyping

DNA was extracted from leukocytes in whole blood by the standard phenol chloroform method, using the Wizard genomic DNA purification kit (Promega Corp., Madison, WI). For the GWAS stage, the samples were genotyped on the Genome-Wide Human SNP Array 6.0 (Affymetrix, Santa Clara, CA). Stringent quality control (QC) procedures were applied to SNP data [e.g., sample-wise and SNP call rate ≥0.95, Hardy–Weinberg equilibrium (HWE) p value of ≥0.001, and minor allele frequency (MAF) of ≥0.05]. The final GWAS data consisted of 534,848 SNPs in 320 subjects used for further analyses.

For the replication stage, genotyping was performed using the ABI PRISM 7900HT Sequence Detection System (Applied Biosystems, Foster City, CA), according to the manufacturer’s protocol. The genotype data for 430 subjects were used for the G × E interaction analysis.

### Expression quantitative trait locus (eQTL) mapping

The Human Genetic Variation Browser (http://www.genome.med.kyoto-u.ac.jp/SnpDB/) is a resource database designed for the investigations on the relationship between genetic variation and gene expression levels in peripheral blood cells in the Japanese population [36]. Eligible donors comprised 291 individuals of both sexes and between 32 and 66 years of age. The DNA genotyping is performed from each donor’s blood sample. In this study, *cis*-eQTL analysis in the Human Genetic Variation Browser was utilized to test our most significant variant for correlation with nearby residing genes’ expression.

### Statistical analyses

The *t*-test was used to compare age, SRRS, CES-D, fearfulness of unemployment, and K6 values between males and females. Comparisons of SRRS or fear of unemployment mean scores among genotype groups were analyzed using analysis of variance (ANOVA). For the GWAS data, a conventional association analysis under an additive model was first performed using linear regression with PLINK ver. 1.07 [37]. Age and sex were adjusted in the analysis because the prevalence of depression is higher in females and the risk of depression increases with age [38, 39]. To account for the genetic substructure of human populations, multidimensional scaling (MDS) was calculated using PLINK and 10 MDS components were also included as covariates in the analyses. Next, the statistical significance of the G × E interaction term was assessed by linear regression model, in which we applied a standard approach to test G × E interaction using the equation: D_ge_ = β_0_ + β_g_G + β_e_E + β_ge_GE. In the model, the depression state (D_ge_: CES-D or K6 scores) was treated as the dependent variable, and the SNP genotype (G), the SLEs (E), the G × E interaction term were treated as independent variables. In the model, sex, age, and 10 MDS components were also included as covariates. When several significant interactions were found in the same region, a conditional analysis was performed to test the independence of each interaction, in which the most significant interaction term was added as an additional covariate in the model. The quantile–quantile (QQ) and Manhattan plots of *p* values from the GWAS stage were generated using qqman R package (http://cran.r-project.org/web/packages/qqman/). The level of genome-wide and suggestive significance was considered to be p < 5.0 × 10^−8^ and p < 1.0 × 10^−5^, respectively. For the G × E interaction, a Bonferroni correction was used to adjust for p-values (p-values were divided by the number of SNPs and an environmental measure). Therefore, the level of genome-wide and suggestive significant interaction was considered to be p < 2.5 × 10^−8^ and p < 5.0 × 10^−6^, respectively. Power calculations were performed using the program Quanto v1.2.4 (http://hydra.usc.edu/gxe). The *t*-test and ANOVA were conducted using the SPSS Statistics 22 for Windows. Statistical tests were 2-tailed and the significance level was set at p < 0.05.

## Results

Since sex and age have been reported to be related with depression [1], we investigated the relationship of depression symptoms and stress events with age and sex. Table 1 represents the demographic characteristics of the samples. For the GWAS stage sample, no significant differences were found in age, SRRS, or CES-D scores between males and females. Distributions of the CES-D scores are presented in S1 Fig. No significant correlation was observed between CES-D scores and age (r = -0.046, p = 0.42). Scores on the SRRS for recent events showed a weak but no significant correlation with depressive symptoms (r = 0.084, p = 0.14). No significant association of SRRS scores with sex was observed, whereas significant correlation with age was found (r = -0.19, p = 0.001). For the replication stage sample, age in males was significantly higher than that in females (t = 2.27, p = 0.024), although no significant differences were observed in K6 values and the assessment of fear of being unemployed between males and females. Distributions of the K6 scores are presented in S2 Fig. A weak but no significant correlation was observed between K6 scores and age (r = -0.097, p = 0.054). Scores on the fear of unemployment showed a significant association with the K6 scores (r = 0.15, p = 0.003), but no significant associations with sex and age were found.

**Table 1:** Demographic characteristics of samples.

	Total	Male	Female	P
Number (GWAS)	320	163	157	
Age ± SD	40.8 ± 9.6	41.6 ± 10.2	39.9 ± 8.7	0.12
SRRS ±SD	53.0 ± 101.0	45.2 ± 51.9	63.5 ± 134.7	0.20
CES-D ± SD	10.8 ± 7.4	10.3 ± 7.9	11.3 ± 6.9	0.27
Number (replication)	439	276	163	
Age ± SD	36.8 ± 8.2	37.5 ± 8.5	35.7 ± 7.6	0.024
fear of being unemployed ± SD	1.04 ± 0.25	1.04 ± 0.26	1.05 ± 0.23	0.49
K6 ± SD	6.2 ± 5.5	6.2 ± 5.7	6.4 ± 5.1	0.75

CES-D: Center for Epidemiologic Studies Depression Scale; SRRS: Social Readjustment Rating Scale; SD: standard deviation.

T-tests were used to compare age, SRRS, CES-D, fear of being unemployed, and K6 values between males and females.

In the GWAS stage, all SNPs passing QC (n = 534,848) were tested for the analyses. We first conducted a conventional GWAS. The QQ and Manhattan plots of the GWAS are illustrated in S3 Fig. The QQ plots of the p values indicate no notable deviation from random expectation (inflation factor λ = 1.003), suggesting no substantial effect of population structure. In the GWAS, no single variant achieved genome-wide significance; suggestive significant associations were found at two SNPs (rs16973084 and rs1514316) on 15q (p = 7.88 × 10^−6^; S1 Table and S3 Fig). These two SNPs are located on the same LD block (D’ = 1.00, r^2^ = 0.46) in the intergenic region. The third strongest significant SNP was rs7016807 (p = 1.03 × 10^−5^) located on *DENN domain-containing protein 3* (*DENND3*) at 8q24.3, of which function is to promote the exchange of GDP to GTP and may play a role in protein transport. SNPs with p-values < 1.0 × 10^−4^ were shown in S1 Table.

**Fig 1:**
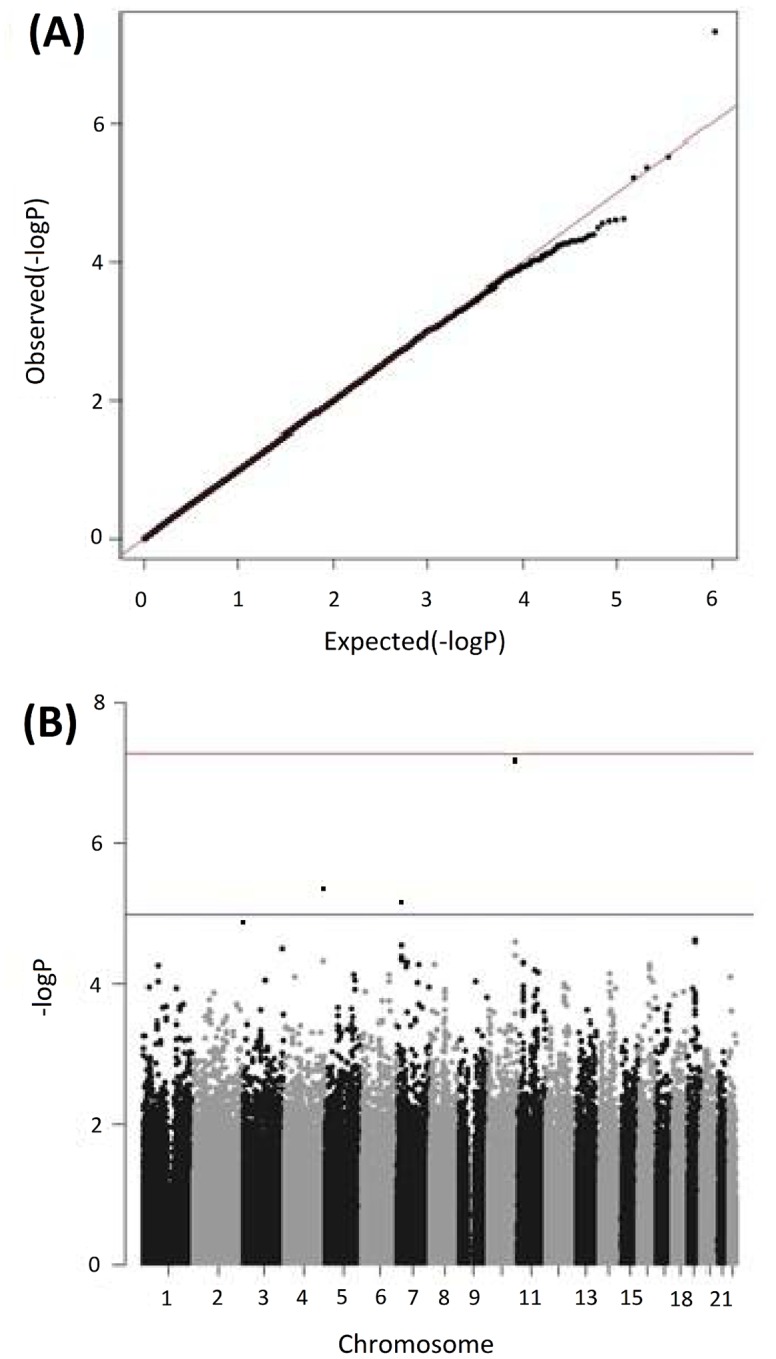
Quantile–Quantile (QQ) and Manhattan plots of genome-wide gene–environment interaction analysis. (A) QQ plots; the observed (−log_10_ *p*) are plotted against the expected (−log_10_ *p*) under no association (diagonal line). (B) Manhattan plots; the (−log_10_ *p*) is plotted according to its physical position on successive chromosomes. The lower and upper horizontal lines represent suggestive significance with *p* < 10^−5^ and genome-wide significance with *p* < 5 × 10^−8^, respectively. Although not significant, a SNP rs10510057 on 10q26 reached marginally genome-wide significance.

**Fig 2:**
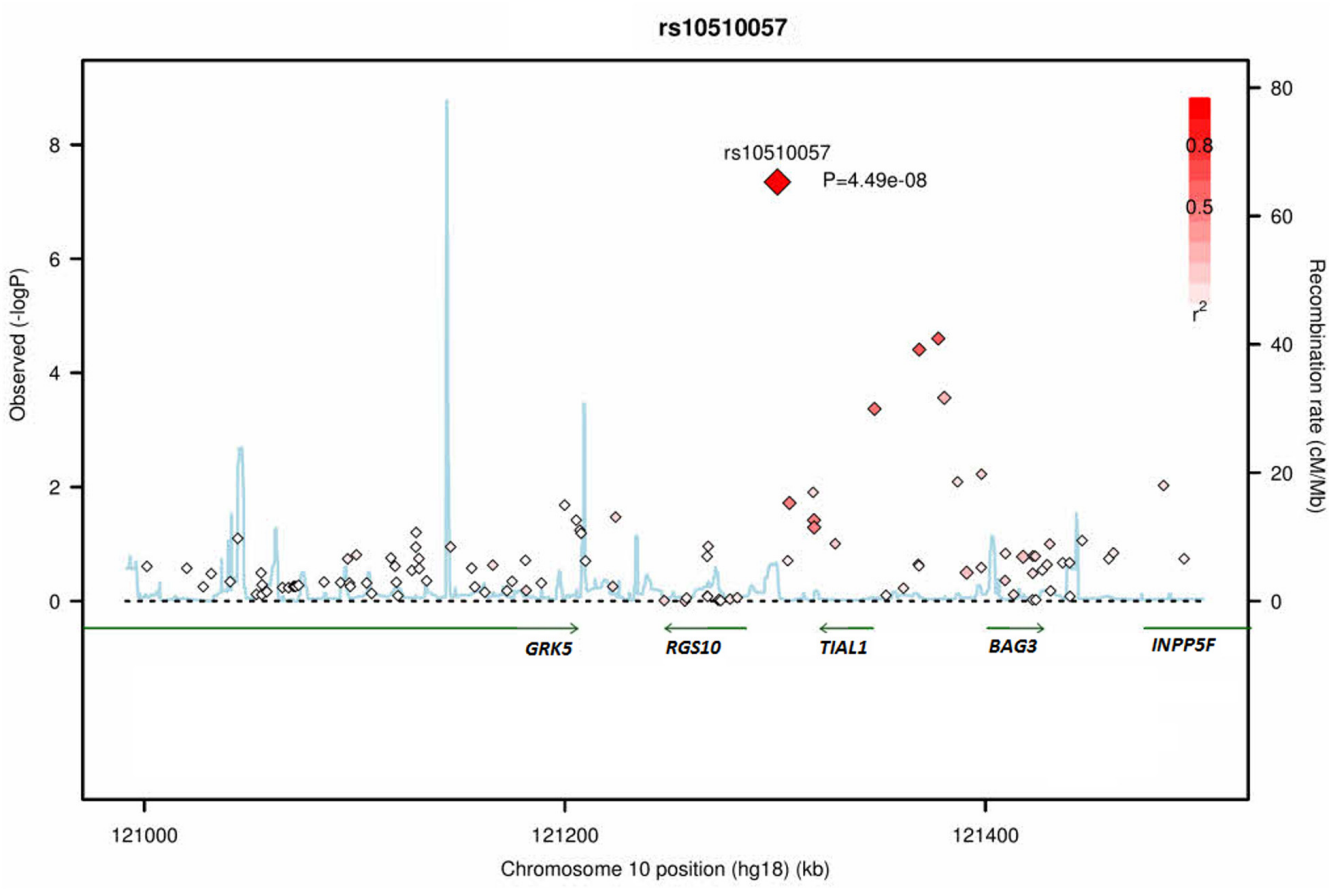
Plots of association results (−log_10_ *p*) at 10p26 region in the genome-wide gene–environment interaction analysis. Chromosome position is plotted according to its physical position with reference to the NCBI build 36. Recombination rate as estimated from the JPT and CHB HapMap data is plotted in light blue. Large red diamond: SNP with strongest evidence for association (rs10510057). Strengths of linkage disequilibrium (LD) (r^2^) with SNP rs10510057 in the plots are shown (dark red indicates stronger LD).

**Fig 3:**
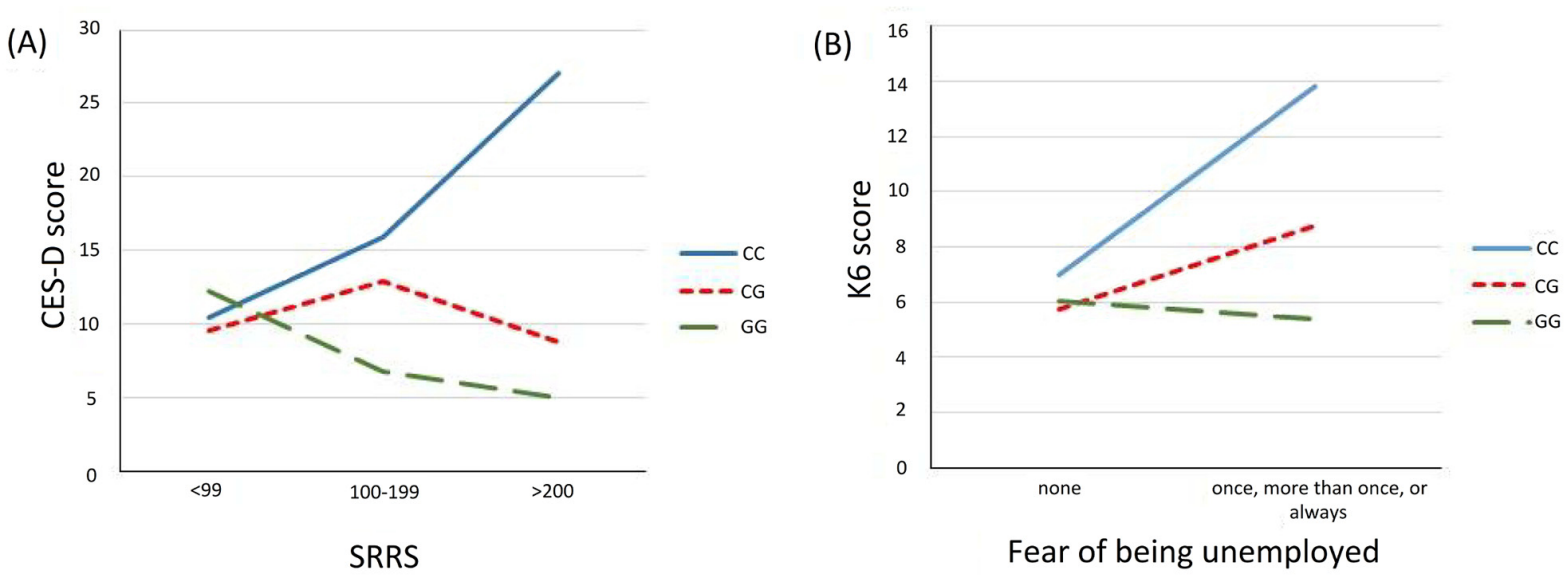
The mean scores of depression symptoms measures grouped by rs10510057 genotypes and exposure to stressful events. (A) CES-D mean scores grouped by genotypes and SRRS scores. (B) K6 mean scores grouped by genotypes and assessments of fear of being unemployed.

Next, we performed a genome-wide G × E interaction analysis. Fig 1 presents the QQ and Manhattan plots of the analysis. The QQ plot shows some but no substantial inflation of association (λ = 1.06). The top SNPs from the G × E interaction analysis are summarized in Table 1 (SNPs with p < 1.0 × 10^−4^ were shown in S2 Table). The marginally genome-wide significant interaction was found at a SNP rs10510057 located near *Regulators of G-protein signaling 10* (*RGS10*) and *TIA1 cytotoxic granule-associated RNA-binding protein-like 1* (*TIAL1*) on 10q26 (p = 4.49 × 10^−8^; Table 2 and Fig 1). Other suggestive interactions (p < 5.0 × 10^−6^) were found at two SNPs located in the intergenic regions (rs13151036 on 4q and rs204595 on 7p; Table 2). Adjacent to the most significant SNP rs10510057, four interactions between SNPs (rs12217200, rs3847487, rs7099126, and rs196267) and SRRS were significant at a value of p < 0.001 (Fig 2), which suggested that the results were not likely to be chance findings. To test the independence of these interactions, a conditional analysis was performed. When each interaction was analyzed controlling for the interaction between rs10510057 and SRRS, none were significant (all p values > 0.05); this shows that the significance of the interaction between each SNP and SRRS in this region was derived from the rs10510057 × SRRS interaction effect. We divided the total SRRS scores into three categories: <99, 100–199, and >200. The mean CES-D scores grouped by rs10510057 genotypes and SRRS categories are depicted in Fig 3. This significant G × E interaction indicated that at higher levels of SLEs, CC carriers presented more depressive symptoms than CG or GG carriers (CC, F = 8.2, p = 0.001; CG, F = 1.7, p = 0.18; GG, F = 2.8, p = 0.070).

**Table 2:** Top findings from genome-wide gene environment interaction analysis.

Chr	SNP	BP	A1	A2	MAF	β	SE	P	Gene
10	rs10510057	121,301,038	C	G	0.489	0.058	0.010	4.49E-08	*RGS10*
4	rs13151036	180,169,855	C	T	0.398	-0.045	0.009	2.82E-06	
7	rs204595	20,877,952	C	G	0.131	0.104	0.022	4.14E-06	
3	rs17193334	1,372,373	A	G	0.065	0.109	0.023	5.80E-06	*CNTN6*
19	rs2607230	33,382,760	G	A	0.106	0.069	0.016	2.29E-05	
19	rs1820708	33,383,865	A	G	0.107	0.068	0.016	2.46E-05	
10	rs7099126	121,377,539	C	T	0.434	-0.042	0.010	2.49E-05	*BAG3*
7	rs6944093	20,767,815	T	C	0.112	0.078	0.018	2.73E-05	*ABCB5*
3	rs6788031	191,859,437	A	T	0.054	0.092	0.022	3.15E-05	*IL1RAP*
10	rs3847487	121,368,483	T	C	0.434	-0.039	0.009	3.89E-05	*TIAL1*
7	rs6944200	20,767,745	A	G	0.111	0.076	0.018	4.10E-05	*ABCB5*
7	rs204585	20,868,813	T	C	0.131	0.085	0.021	4.46E-05	
4	rs1451430	180,165,628	C	T	0.443	-0.039	0.010	4.71E-05	
11	rs10834377	24,521,470	T	A	0.194	0.044	0.011	4.81E-05	*LUZP2*
7	rs12701976	42,474,899	C	A	0.250	0.040	0.010	4.85E-05	

Chr: chromosome; SNP: single nucleotide polymorphism; BP: base position; MAF: minor allele frequency; SE: standard error.

A1: tested allele (minor allele); A1, other allele.

Genes with SNPs located up to 50kb down- or upstream were shown.

P-values are not corrected by Bonferroni correction.

Among SLEs, work-related stress has been reported to be associated with depression [24]. Therefore, we investigated a similar interaction between rs10510057 and a work-related stress in depression in an independent sample of 439 subjects. As a result, a significant G × E interaction effect on depression was found (β = 2.9, p = 0.015). When the assessment of fear of being unemployed was categorized into two variables (“none” vs. “once,” “more than once,” or “always”), the stressor significantly affected the expression of depression in CC carriers (F = 7.7, p = 0.007; Fig 3).

We also investigated the possible association of rs10510057 with the nearby residing genes’ (*RGS10* and *TIAL1*) expression in the Human Genetic Variation Browser. We could not find the results of rs10510057 in the database. Instead, a SNP rs7087527 on the same LD block with rs10510057 (D’ = 1.00, r^2^ = 0.76) showed a significant association with RGS10 gene expression (p = 4.3 × 10^−4^) but not with TIAL1 gene expressions (p = 0.57).

## Discussion

To date only a limited number of candidate gene G × E studies have been conducted for depression. Therefore, a systematic search for G × E interactions using a genome-wide approach is needed. To our knowledge, this is the first pilot study investigating G × E interactions in depression at the genome-wide level. Using a sample of 320 healthy Japanese subjects, we found a marginally genome-wide significant interaction between SLEs and a SNP rs10510057 near *RGS10* and *TIAL1* located on 10q26 although the association did not reach statistical significance. We found that stressful events significantly affected the expression of depression in CC carriers. The conditional analysis revealed that the significance was derived from the interaction between rs10510057 and SRRS. As work-related stress has been reported to be associated with depression, a similar G × E interaction between rs10510057 and work-related stress was investigated in an independent sample of 439 subjects, which also showed a significant association.

Most previous G × E studies assessed depression symptoms in community samples. Assuming that depression is a continuous trait permits the use of all available information; therefore, the statistical power is increased. It has been estimated that a sample of 10,000 is required to detect a moderately strong G × E interaction at a genome-wide level of significance [40]. Our study investigated a relatively small sample (n = 320) and the statistical power was calculated as 0.02% with a genome-wide significance threshold of p < 5 × 10^−8^, and the G × E interaction accounting for 1% of the variance. We need at least 3,500 subjects to attain 80% of power to detect a genome-wide significant signal on this condition. However, collecting of large samples is demanding and is possible at the expense of phenotype homogeneity and precise measure of environmental exposure. In this sense, we consider that each of our study samples was genetically and culturally homogenous as the participants were all Japanese white-collar workers in a company. Furthermore, a GWAS study in smaller samples may reveal important associations, particularly, when the phenotype and environmental variables are assessed systematically and intensively [41]. Therefore, the suggestive findings in this study may be of value for future studies with larger sample sizes.

In the GWAS stage, we assessed self-reported SLEs that occurred in the past 12 months and encompassed environmental exposures as diverse as relationship, financial, and unemployment stressors. Depression has been reported to be particularly associated with major life events that are characterized by loss, threat, and humiliation [42, 43]. On the other hand, other fields of SLEs (e.g., relationship problems with a close friend, housing problems, and unemployment) are considered to be “minor” rather than “major.” [44] In addition, both acute (e.g., sudden death of a family member) and chronic stressors (e.g., ongoing financial difficulties) are associated with depression. While acute stressors have an impact on illness within a brief period (i.e., approximately a month), chronic stressors lasting 6 months or more cause longer stress symptom episodes and contribute to a greater recurrence rate [45]. These different kinds of SLEs and stressors may have different effects on depressive symptoms. In the replication stage of G × E interaction analysis, we assessed only a single stressor (i.e., “fear of being unemployed”), which may be characterized as a “chronic” and “minor” stressor. Thus, although this was not a strict replication study for “acute” and “major” life events assessed by the SRRS, it supported the G × E effect on depression. However, a strict replication study in future G × E interactions studies should use systematic and intensive assessment of the same key environmental exposures evaluated in the GWAS stage.

We found a suggestively significant interaction between SRRS and rs10510057 located 8kb upstream of *RGS10* on 10q26. The eQTL analysis suggested a strong association between rs10510057 and the RGS10 gene expression level, which was consistent with the evidence that *cis*-eQTLs were more likely to be located in gene structure and the adjacent regions; the majority (70%) of *cis*-eQTL were located within 17kb-flanking or within a target protein coding gene [36]. RGS10 belongs to a subfamily of regulator of G-protein signaling (RGS) proteins, attenuating G-protein-coupled receptor signal transduction of dopamine, serotonin, and glutamate receptors [46, 47]. RGS10 is abundantly expressed in a broad range of brain regions including the dorsal raphe, cortex, and striatum [48]. However, the role of RGS10 protein in the human brain remains unknown. RGS10 localizes in the plasma membrane and its phosphorylation by cAMP-dependent protein kinase A (PKA) has been proposed as a mechanism for translocation of RGS10 from the plasma membrane into the nucleus; this has been suggested as a potential mechanism of regulation of intracellular signaling [49]. Recent studies have shown that RGS10 is regulated by psychotropic drug and stress [50, 51]. Using cadaveric human brains, Rivero et al. found that the immunoreactivity level of RGS10 protein was decreased in the prefrontal cortex of short-term opiate abusers [50]. Polymorphisms in *RGS10* were reported in a cohort of Japanese schizophrenia patients but with negative association data [52]. Pinheiro et al. conducted an association study of 182 candidate genes for anorexia nervosa and found that rs10510057 in RGS10 suggestively associated with the disorder [53]. These reports highlight RGS10 as a promising candidate for stress-related disorders that is worthy of additional follow-up.

Several limitations of this study should be addressed. First, depressive symptoms were measured by self-administered assessment questionnaires. There may be a response bias, resulting in over- or underestimation of the true association. Second, our subjects were employees in two individual companies who would presumably be healthy; therefore, depressive symptoms are not normally distributed. Many of the subjects fell under a peak at the lower end of the score [i.e., low depressive symptoms score (CES-D ≤ 16 or K6 ≤ 9); S1 and S2 Figs]. Thus, because of this particular distribution, *p* values for some markers in GWAS may have been biased. Third, because our main aim was to identify SNPs conferring risk of depression, we did not assess the impact of epigenetic changes. Recent research indicates that epigenetic (e.g., DNA methylation) changes caused by environmental stress may be associated with risk for depression [54]. Therefore, investigating epigenetic modifications on the genomic regions identified in the present study may reveal causal variants associated with depression.

In conclusion, our findings suggested that a SNP rs10510057, interacting with stressful events, may be a risk factor for depression. In addition, incorporating G × E interaction into GWAS confers a greater detection power for identifying susceptibility variants that would be missed in conventional analyses. Further studies are necessary to confirm our findings and clarify the underlying mechanisms of depression.

## Supporting Information

S1 Fig Distributions of the CES-D scores. (TIF)

S2 Fig Distributions of the K6 scores. (TIF)

S3 Fig Quantile-quantile (QQ) and Manhattan plots of genome-wide association study. (a) QQ plots (b) Manhattan plots.
(TIF)

S1 Table Top findings from genome-wide association study (P < 1.00 × 10^−4^). (XLSX)

S2 Table Top findings from genome-wide gene environment interaction analysis (P < 1.00 × 10^−4^). (XLSX)
