# Supplementary figures and images for: The First Pilot Genome-Wide Gene-Environment Study of Depression in the Japanese Population
Source: PLoS One. 2016 Aug 16;11(8):e0160823. doi: 10.1371/journal.pone.0160823 (PMC4986946; doi:10.1371/journal.pone.0160823)

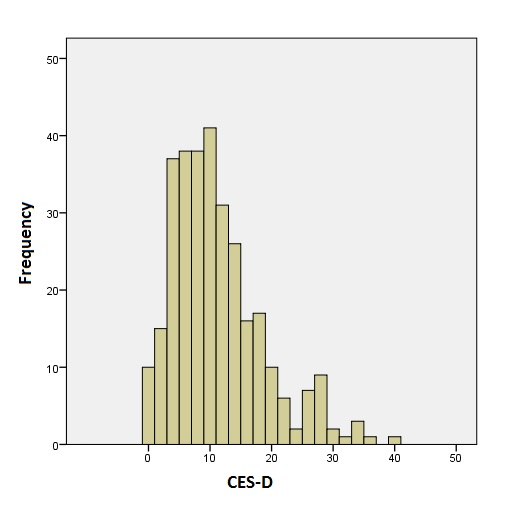

Supplement: S1 Fig — (TIF) [file pone.0160823.s001.tif]

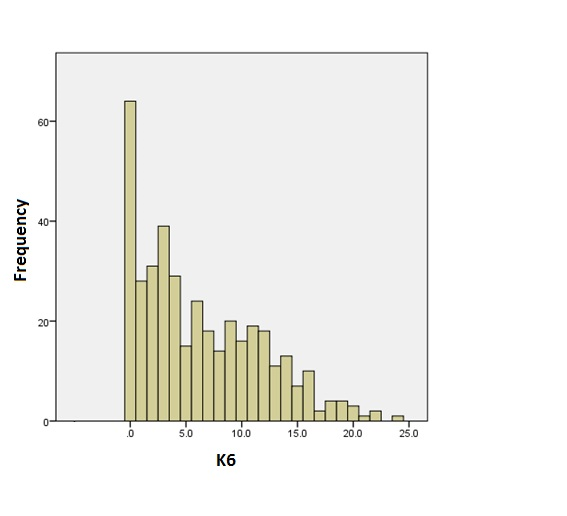

Supplement: S2 Fig — (TIF) [file pone.0160823.s002.tif]

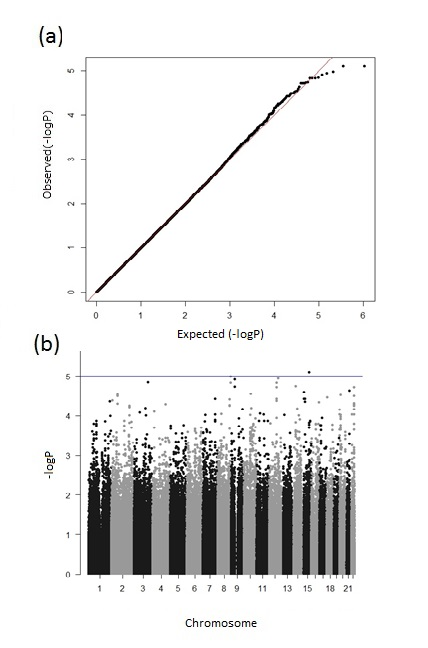

Supplement: S3 Fig — (a) QQ plots (b) Manhattan plots. (TIF) [file pone.0160823.s003.tif]
